# Evaluating the Contribution of Growth, Physiological, and Ionic Components Towards Salinity and Drought Stress Tolerance in *Jatropha curcas*

**DOI:** 10.3390/plants9111574

**Published:** 2020-11-13

**Authors:** Muhammad Mohsin Abrar, Muhammad Saqib, Ghulam Abbas, Muhammad Atiq-ur-Rahman, Adnan Mustafa, Syed Atizaz Ali Shah, Khalid Mehmood, Ali Akbar Maitlo, Mahmood-ul-Hassan, Nan Sun, Minggang Xu

**Affiliations:** 1 National Engineering Laboratory for Improving Quality of Arable Land, Institute of Agricultural Resources and Regional Planning, Chinese Academy of Agricultural Sciences, Beijing 100081, China; randhawasab2016@gmail.com (M.M.A.); adnanmustafa780@gmail.com (A.M.); syedatizaz.shah@gmail.com (S.A.A.S.); aamaitlo@gmail.com (A.A.M.); xuminggang@caas.cn (M.X.); 2 Institute of Soil and Environmental Sciences, University of Agriculture, Faisalabad 38040, Pakistan; g.a92pk@gmail.com (G.A.); sardaratiq971@gmail.com (M.A.-u.-R.); 3 Department of Environmental Sciences, COMSATS University Islamabad, Vehari Campus, Punjab 61100, Pakistan; 4 Soil Survey of Punjab Lahore, Lahore 54780, Pakistan; 5 Collaborative Innovation Center on Forecast and Evaluation of Meteorological Disasters (CIC-FEMD), Key Laboratory for Aerosol-Cloud-Precipitation of China Meteorological Administration, School of Atmospheric Sciences Nanjing University of Information Science and Technology, Nanjing 210044, China; sipra.khalid770@gmail.com; 6 Agriculture Department, Soil Fertility Research Institute, Government of Sindh, Tando Jam 70060, Pakistan; 7 Department of Plant Nutrition, College of Resources and Environmental Sciences, China Agricultural University, 2 W Yuanmingyuan Ave, Haidian, Beijing 100193, China; hassanagr17@gmail.com

**Keywords:** *Jatropha curcas* L., salinity, drought stress, stress tolerance, biomass, chlorophyll content, ionic composition

## Abstract

Salinity and drought stress, singly or in combination, are major environmental menaces. *Jatropha curcas* L. is a biodiesel plant that can tolerate long periods of drought. However, the growth performance and stress tolerance based on physical, chemical, and physiological attributes of this plant have not yet been studied. To address this question, *J. curcas* seedlings were grown in a completely randomized design in plastic pots filled with soil to evaluate the effects of salinity and drought stresses on growth, ionic composition, and physiological attributes. The experiment consisted of six treatments: control (without salinity and drought stress), salinity alone (7.5 dS m^−1^, 15 dS m^−1^), drought, and a combination of salinity and drought (7.5 dS m^−1^+ Drought, 15 dS m^−1^+Drought). Our results revealed that, compared with the control, both plant height (PH) and stem diameter (SD) were reduced by (83%, 80%, and 77%) and (69%, 56%, and 55%) under salinity and drought combination (15 dS m^−1^+Drought) after three, six, and nine months, respectively. There was 93% more leaf Na^+^ found in plants treated with 15 dS m^−1^+Drought compared with the control. The highest significant average membrane stability index (MSI) and relative water content (RWC) values (81% and 85%, respectively) were found in the control. The MSI and RWC were not influenced by 7.5 dS m^−1^ and drought treatments and mostly contributed towards stress tolerance. Our findings imply that *J. curcas* is moderately tolerant to salinity and drought. The Na^+^ toxicity and disturbance in K^+^: Na^+^ ratio were the main contributing factors for limited growth and physiological attributes in this plant.

## 1. Introduction

The extreme events associated with global climate change jeopardize sustainable plant growth and production worldwide [1]. The changes in abiotic constraints such as salt stress, drought, and temperature are typical examples of such alterations. Among abiotic stresses, soil salinization and drought extremes have been regarded as leading stresses limiting agricultural production. Given that efforts had been made in the past to ameliorate the negative impacts of these environmental constraints on plant growth [2,3,4], only a few studies are available to date that explore the combined effects of salinity and drought stresses on *Jatropha curcas* [5].

Salinity and drought stresses generally occur concurrently [6]. The increase in dryland areas is often associated with the consumption of low-quality irrigation water. Thereby, the continuous progression of drought and salinity stress results in a decrease in the available arable land for agriculture, especially in arid to semi-arid regions of the world [7]. 

Salinity and drought are two major environmental constraints for crop productivity and are consistently threatening agricultural systems [8]. Salinity affects approximately 50% of irrigated lands worldwide [9].

Soil salinity causes ion accumulation at toxic levels and osmotic stress in plants, resulting in growth and developmental reductions [10], which causes lower soil matric potential and leads to drought conditions, i.e., those that cause physiological limitation for plant growth [10]. It has been established that halophytes can survive under high salt concentrations (>200 mM NaCl), while glycophytes can only sustain their growth under relatively low salinity conditions [11,12]. Stunted plant growth and low crop yields are the two foremost impressions of salinity and drought stresses. Therefore, it becomes imperative to introduce economically efficient, environmentally friendly, and sustainable management approaches to tackle such environmental constrains to ensure food security. 

Stress tolerance (i.e., salinity or drought) can be defined as the ability of the plant to sustain sufficient growth under stress milieu [10]. More precisely, the salinity tolerance can be termed as producing biomass on a percent basis in controls in contrast to saline treatments for a longer time duration. Several attributes are associated with the physiological and developmental mechanisms of plants such as the buildup of osmotically active solutes, osmotic adjustment, and a partial stomatal opening for tolerating long episodes of drought stress [13]. The closing of stomata is a double-edged sword, i.e., it decreases C uptake on one hand while minimizes water loss on the other hand. Hence, plants sustain osmotic adjustments in tissues under salinity and drought stress conditions. Such a type of benefit (efficient water consumption) is more significant in C_4_ plants than C_3_ plants [14].

*Jatropha curcas* (physic nut) is a C_3_ shrubby plant (family *Euphorbiaceae*) [15], adaptable to marginal soils, and is capable of ameliorating problematic soils and retrieve soil productivity [16]. In recent times, *Jatropha* has gained the exceptional attention of the scientific factions because of its bioenergy, salinity [17], and drought-resistance [18] in various agricultural settings. 

Little is known about the combined effect of salinity and drought stresses on the growth performance of *Jatropha*. Conversely, it is well known that *Jatropha* is drought-resistant, since many scientists have quantified the performance of Jatropha under water deficit conditions. For example, Maes et al. [17] demonstrated that drought considerably reduced plant growth; however, it did not affect physiological parameters (water relations). Moreover, Achten et al. [19] have examined that biomass production of *Jatropha* was 57% more in well-watered conditions compared with medium water supply (40% plant available water). Nevertheless, variations are found regarding the imposition of drought stress in various studies, and the interaction of both salinity and drought stresses has not been well quantified. Moreover, *Jatropha* had demonstrated sufficient biomass production under the stressful environment of semi-arid areas and its overall performance to salinity and water deficit conditions has been explored. However, its stress tolerance based on biomass production, ionic composition, and physiological mechanisms under the interaction of salinity and drought is not well described. Taking this context into account, we hypothesized that combined salinity and drought stresses may affect growth and development of *J. curcas* while their effects are further governed by stress tolerance ability of *J. curcas*.

Therefore, the current study was carried out to unravel the effects of salinity and drought stresses on growth, ionic composition, and physiological attributes of *J. curcas*. 

## 2. Results

### 2.1. Growth Parameters Affected by Salinity and Drought

Salinity, drought, and their interaction significantly affected plant height (PH) and stem diameter (SD) (*p* < 0.05; Table 1). Compared with the control, both the PH and SD were reduced with increasing salinity and drought. The maximum decrease (83%, 80%, and 77%) in the PH was observed at an interactive level of salinity and drought (15 dS m^−1^+Drought) after three, six, and nine months, respectively, compared with the control. Simultaneously, a similar decreasing pattern (69%, 56%, and 55%) in SD was recorded after three, six, and nine months, respectively. The plants died at 22.5 dS m^−1^ and 22.5 dS m^−1^+Drought, due to extreme conditions. 

Salinity and drought stress significantly (*p* < 0.05) affected both the number of branches (NOB) and number of leaves (NOL). The maximum NOB and NOL were found in the control, while the least values for both parameters were observed in 15 dS m^−1^+Drought. The NOB averaged from 4.80, 5.20, and 7.60 after three, six, and nine months, respectively. The NOL followed a similar trend at the same level (*p* < 0.05; Table 1). 

**Figure 1:**
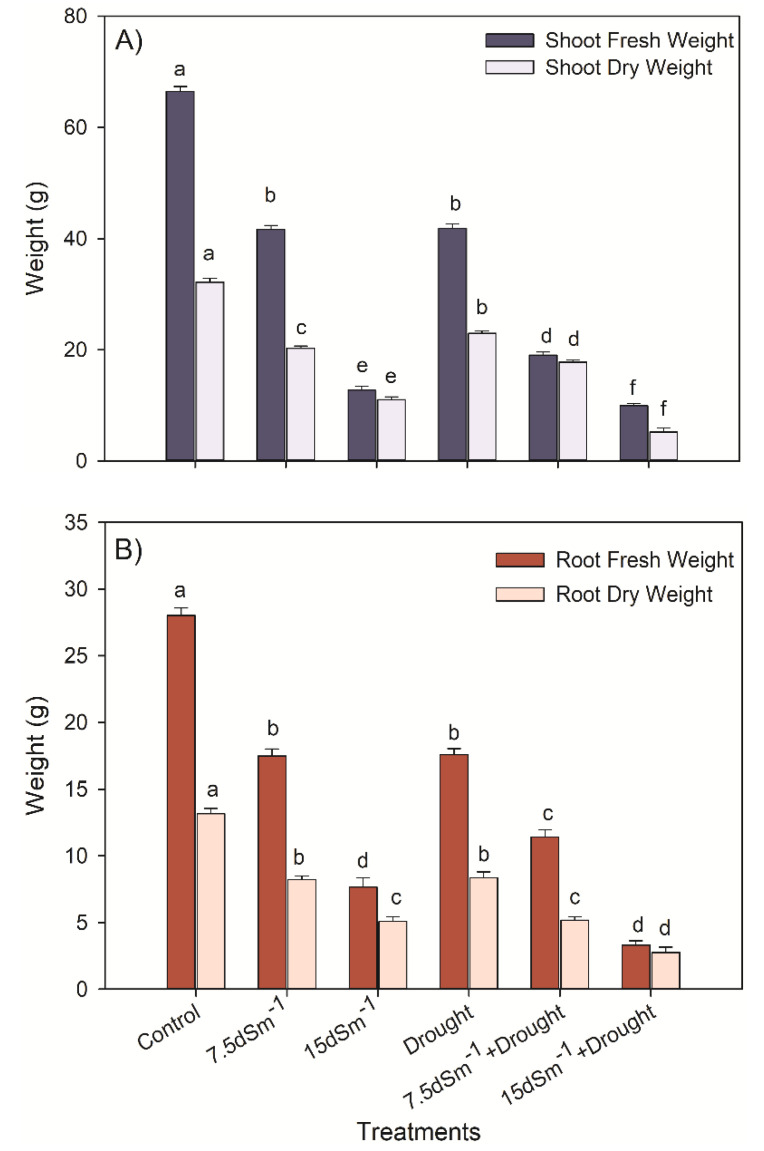
(**A**) Shoot fresh weight (SFW) and dry weight (SDW), and (**B**) root fresh and dry weight (*p* < 0.05), following post-hoc Duncan’s multiple range test.

Compared with the control, the SFW and SDW were significantly decreased under 15 dS m^–1^+Drought (*p* < 0.05; Figure 1). The highest SFW and SDW values (66.48 and 32.13 g plant^–1^) were recorded in the control. At the same time, the maximum decrease of 85% and 84% in the SFW and SDW, respectively, was noted at the 15 dS m^–1^+Drought in contrast to the control. A trend similar to the SFW and SDW was also found in the RFW and RDW subjected to 15 dS m^–1^ and 15 dS m^–1^+Drought (Figure 1B). In the control, the maximum mean values (28.02 and 13.16 g plant^–1^) of the RFW and RDW, respectively were noted. Whereas no significant difference was observed in the RDW values of plants subjected to 7.5 dS m^–1^+Drought (250 mL) treatments. The highest reduction in the RFW and RDW of 88% and 79% was noted at 15 dS m^–1^+Drought in contrast to the control.

**Figure 2:**
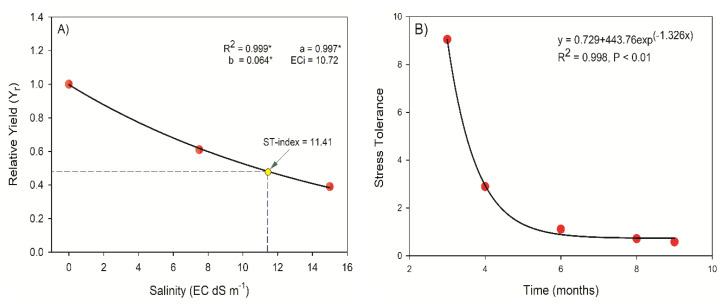
(**A**) The effect of salinity on the plant yield (in terms of total dry mass). The yellow circle shows the ST-index, and the ECi is the electrical conductivity of the irrigation water at the yield reduced to 50% of the absolute yield (Y). Experimental data (dots). The model curve for Yr = a*eb*ECi. EC_i50_ and ST-index are the mid-yield salinity and the salinity tolerance index, respectively, as calculated by Steppuhn et al. (2005). * indicates significant differences at *p* < 0.05. (**B**) Exponential decay model fitted on the growth-related parameters, such as plant height, stem diameter, and total dry mass-produced (adapted from Fernandez [20] with slight modifications).

Figure 2A presents the yield of *J. curcas* corresponding to salinity and a relative decline in relative biomass that is inversely proportional to salinity is apparent. The values of electrical conductivity in the irrigation of a water reduced yield that was 50% of the maximum yield (EC_i50_) and the salinity-tolerance index (ST-index) were 10.72 dS m^−1^ and 11.44 dS m^−1^, respectively, implicating a low vulnerability of *J. curcas* to salinity. A relationship was developed between the stress tolerance of *J. curcas* plants and time on the basis of the exponential decay model, A = Ao + ae^kt^, where "a" is intercept "k" is the decay rate. Stress tolerance first decreased exponentially till six months and then attained an asymptotic relationship (Figure 2B).

### 2.2. Ionic Concentrations

The results of this study indicate that marked variations in Na^+^ concentration in the shoot were observed in 15 dS m^−1^+Drought compared with the control. Consistent with the shoot Na^+^ results, the root followed the same trend, and the maximum Na^+^ concentration in root and stem were determined as 2.33 and 2.07 mmol g^−1^ dw, respectively, under 15 dS m^−1^+Drought treatment. The lowest leaf, stem, and root Na^+^ mean values (0.12, 0.14, and 0.16 mmol g^−1^ dw, respectively) and highest K^+^ mean concentrations (1.62, 0.68, and 0.55 mmol g^–1^ dw, respectively) were recorded in the control followed by the drought treatment. Contrary to the trend in the control, the highest mean Na^+^ (1.84, 2.06 and 2.32 mmol g^−1^ dw) and lowest K^+^ values (0.75, 0.36, and 0.27 mmolg^−1^ dw) in leaf, stem, and root, respectively, were recorded in the 15 dS m^−1^+Drought treatment (Table 2).

The highest K^+^: Na^+^ ratios (13.06, 4.72, and 3.49) in leaf, stem, and root were found in the control, whereas the lowest K^+^: Na^+^ mean values were noted in 15 dS m^−1^+Drought. The Drought treatment followed the control and showed 7.95, 3.28, and 2.53 of K^+^: Na^+^ ratios in leaf, stem, and root, respectively. However, no significant (*p* > 0.05) difference in K+: Na^+^ ratios of stem and root were found among 15 dS m^−1^, 7.5 dS m^−1^+Drought and 15 dS m^−1^+Drought treatments (Table 2).

### 2.3. Physiological Parameters and Water Relations

A significantly highest average value (81%) of MSI was found in the control (*p* < 0.05; Figure 2A), whereas the smallest value (44%) of MSI was noted in 15 dS m^−1^+Drought among all treatments. The combination of salinity and drought (15 dS m^−1^+Drought) caused the highest decrease (by 49%) in MSI. However, 7.5 dS m^−1^+Drought treatments did not cause a notable effect on the MSI (*p* > 0.05).

Like MSI, the RWC was notably higher (*p* < 0.05) in the control compared to the other treatments except 7.5 dS m^−1^+Drought treatments (Figure 2B). The highest RWC value (85%) was found in the control, followed by the drought treatment (i.e., 77.2%). However, these were statistically non-significant. With increasing salinity, the RWC was significantly reduced in both treatments, i.e., 15 dS m^−1^ and 15 dS m^−1^+Drought, and showed the lowest values, i.e., 51.6%, and 53.2% of RWC values, respectively.

The photosynthetic rate (PR) differed significantly (*p* < 0.05) among the treatments. For example, in the control, the highest PR (6.25 µmol m^−2^sec^−1^) was observed, while the lowest PR was determined in 15 dS m^−1^+Drought. The most significant decrease (73%) in the PR was noticed at 15 dS m^−1^+Drought in comparison to the control. Similarly, the highest transpiration rate (TR) (1.98 mmol m^−2^sec^−1^) was determined in the control. At the same time, the lowest TR was found in 15 dS m^−1^+Drought.

In the control, the highest mean stomatal conductance (0.64 mmol m^−2^ sec^−1^) was noted among all the treatments. Whereas the maximum decrease (87%) in the stomatal conductance was observed in 15 dS m^−1^+Drought. The highest mean value, i.e., 25 (in SPAD units) of chlorophyll content, was found in the control. The maximum decrease of 80% was observed in 15 dS m^−1^+Drought compared with the control, followed by 15 dS m^−1^ of (68%), respectively.

## 3. Discussion

In broad terms, salinity stress can be composed of two components: osmotic stress and salt specific toxicity (Na^+^ toxicity and ionic imbalance). The combined effect of salinity and drought (in the present study) had a considerable influence on *Jatropha*’s growth and development (Table 1).

Following the EC_i50_ and ST-indexes (10.72 dS m^−1^; 11.41), *J. curcas* seems to be more resistant to salinity (Figure 2A) than the other crops, e.g., the almond (ECi_50_ = 3.83 dS m^−1^, ST-index = 4.94), apricot (EC_i50_ = 3.39 dS m^−1^, ST-index = 4.63) given by Steppuhn et al. [21]. 

### 3.1. Growth Parameters Affected by Salinity and Drought

The growth reduction of plants under salinity and water deficit conditions (drought) is a common finding in the present study, and similar results are also reported by the other studies [22,23,24]. The present study demonstrated that the salinity negatively affects the PH and SD and may be attributed to the decline in osmotic potential and nutrient limitation [25]. Moreover, decreased osmotic potential resulted in the closing of stomata and the deactivation of the enzyme-associated system and eventually reduced the plant growth. Moreover, reduced CO_2_ fixation and N assimilation induced a depreciation in the plant growth and reduced the PH. Simultaneously, excessive salt ions in the cell wall manipulated the metabolism and, therefore, decreased the cell wall’s elasticity and consequently limited the PH [26].

*Jatropha* can sustain its growth in rainfall conditions ranging from lowest (i.e., 200 mm) to highest (1200 mm) [27]. For example, our findings suggest a 46%, 47%, and 47% reduction in PH after three, six, and nine months, respectively, in plants irrigated with 40% of soil water holding capacity (WHC) (i.e., drought stressed) compared with those irrigated with 70% of soil WHC (well-watered) (Table 1).

The type of plant, plant organ, and growth stages determine the considerable variability regarding salinity impact [12]. Moreover, Díaz-López et al. [28] and Yaron et al. [20] explored whether increasing salinity reduced stem diameter and leaf growth and hence attained less weight, number of branches, and leaves. Other studies reported a decrease in SD subjected to different salinity regimes [4,29]. This reduction in SD may be attributed to the decline in the turgor potential [28]. Similarly, we reported a decrease in SD for instance (by 69%) after three months in plants treated with combined salinity and drought stresses (15 dS m^−1^+Drought) compared with the control. 

In line with past studies [23,24], our findings indicated a decrease in plant biomass and SFW with increasing salinity and drought. Salinity reduces biomass production and growth in many plant species, and those plants that able to produce extra dry mass can survive for a longer time in saline plus drought conditions [25]. Furthermore, those plants are more salt-tolerant and are capable of excluding Na^+^ from shoot to roots [30].

In contrast to non-saline conditions, salinity negatively impacts the SFW and SDW, which can be proposed as an indication for salinity tolerance at initial growth phases of the plant [31], and excessive Na^+^ concentrations are considered degrading for plant growth [32]. This implies that salt stress slows down the metabolic mechanisms and leads to decreased growth, and consequently, a reduction in biomass occurs [33]. 

In our study, in contrast to the control, salinity (7.5 dS m^−1^) decreased RFW and RDW by 45% and 43%, respectively. However, drought treatment did not affect so severely, as 7.5 dS m^−1^ affected both RFW and RDW (Figure 1A). Moreover, combined salinity and drought treatments influenced the root growth relatively more adversely; for instance, in an experiment, Soda et al. [34] reported that salinity reduced the root architecture in the olive plant. A similar pattern was given by Bernstein et al. [35], who concluded that avocado root growth compared with shoot growth might be more sensitive to salinity.

A large amount of leaf and root litter influence the physicochemical characteristics of soils [36]. The rhizodeposition is a vital energy reserve for microbes (in the form of sucrose or starch, etc.), which alternatively regulates microbial activity [37]. Saline stress alone or in combination with drought considerably (*p* < 0.05; Figure 1B) reduces root fresh and dry weights [38,39]. Similarly, Carillo et al. [40] proposed that salinity depreciated fresh and dry biomass. 

### 3.2. Chemical Parameters Affected by Salinity and Drought

**Figure 3:**
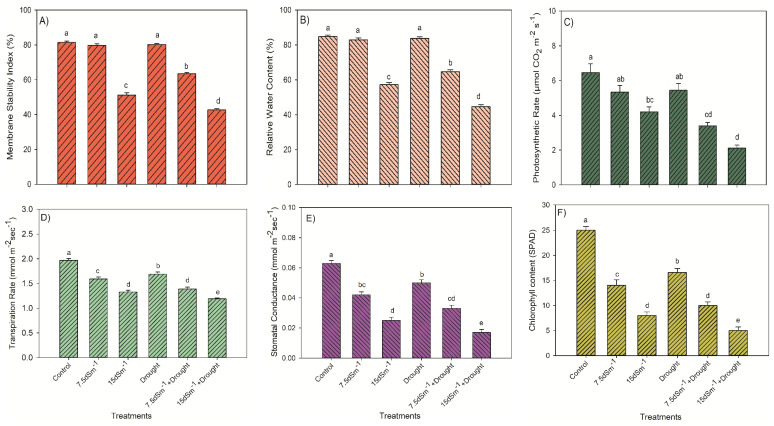
Effect of different levels of salinity and drought on the (**A**) membrane dtability index (**B**) relative water content (**C**) photosynthetic rate (**D**) transpiration rate (**E**) stomatal conductance (**F**) chlorophyll content. Different letters depict a significant difference at *p* < 0.05.

Plants tend to maintain high K^+^ concentration in contrast to Na^+^ in stem and root. Numerous researchers have concluded that plants tend to attain sufficient K^+^ levels that might effectively diffuse the toxic effects of Na^+^ in plant tissues [41,42,43]. In comparison, the elevated leaf Na^+^ concentration corresponding to the lowered leaf K^+^ concentration under salt treatments results in K^+^: Na^+^ ratio decline. We found that K^+^: Na^+^ ratio decreased with an increasing salinity level. Moreover, potassium is a necessary macro-nutrient responsible for the activation of more > 50 enzymes [44] inclusive of the enzymes which participated in the biosynthesis of chlorophyll. Both Na^+^ and K^+^ ions have a very similar ionic radius and hydration energy. Therefore, under saline conditions, Na^+^ enters the cell by using K^+^ channels located at cell membranes [45]. The higher cytoplasmic concentration of Na^+^ leads to a lower K^+^: Na^+^ ratio, which ultimately affects plant metabolism. Hence, the capability of the plants to limit K^+^ loss and maintenance of high ionic ratio (K^+^: Na^+^) in the cytoplasm is an indication of their salt tolerance potential [46]. In the present study, we noticed a considerably higher K^+^: Na^+^ ratio in the control, as compared with the rest of the treatments, and produced more biomass (Table 2). For instance, in comparison to the control, maximum decrease (86%) in biomass was noted in combination of salinity and drought (15 dS m^−1^+Drought), whereas the lowest decrease (37%) was recorded in Drought treatment. The lowest K^+^: Na^+^ ratio at 15 dS m^−1^+Drought treatment also affected physiological parameters adversely, for example, it reduced the photosynthetic rate, chlorophyll contents, stomatal conductance, and transpiration rate by 67%, 80%, 73%, and 40%, respectively, compared with the control (Figure 3).

### 3.3. Physiological Parameters Affected by Salinity and Drought

Our findings recorded a 49% and 53% reduction in the MSI and RWC, respectively, in 15 dS m^−1^+Drought in comparison to the control; however, no statistical difference (*p* > 0.05) was observed in the MSI and RWC among the control, 7.5 dS m^−1^ and Drought treatments (Figure 2A,B). In present study, our results validated that the MSI and RWC were the major attributes contributing towards stress tolerance, since these were less affected by salinity (7.5 dS m^−1^) and water deficit (Drought). However, with increasing salinity and under the combined effect of salinity and drought, both of these parameters were influenced significantly. Similarly, Kotula et al. [47] noticed a 40% and 33% reduction in the MSI and RWC, respectively, due to salinity stress in comparison to the control in melon. Salinity-induced effects can serve as an essential indicator for water relations to quantify the salt tolerating ability of plants [48]. In our study, a greater amount of biomass was recorded in the combination of low salinity and drought (7.5 dS m^−1^+ Drought) compared with the high salinity treatment (15 dS m^−1^) alone. It was further validated by the high value of the RWC in the same treatment.

The RWC has been proposed as an easy agricultural attribute to select plants for their tolerance to salinity, drought, and heavy metal contamination on the basis of a high RWC [49]. Salinity and drought are responsible for causing manipulations in MSI, which may indicate cell damage [50]. The ability of plants to maintain normal transpiration rates under stress conditions reflects their stress tolerance, since transpiration is often associated with the normal assimilation of CO_2_ for photosynthesis [51].

Chlorophyll is a green-colored pigment responsible for the vital process of photosynthesis to make food [52], and the chlorophyll content can be assessed in terms of SPAD units, which is likely to decrease in saline conditions compared with the control. The relative decrease in SPAD units was mainly driven by genetic control [53]. Under stress conditions (i.e., salinity and drought), the relative salt accumulation and the stress-tolerating ability of the plant determine the reduction in chlorophyll contents [54]. Additionally, Velagaleti et al. [55] concluded that the decrease in chlorophyll contents was ascribed to chloride accumulation (Cl^−^). Moreover, Rangani et al. [56] concluded that in quinoa, the decline in chlorophyll contents was mainly attributed to the disintegration of chlorophyll structure under higher salinity levels.

Generally, in most plants, salt stress reduces the photosynthetic rate [57]. Furthermore, Yang et al. [58] reported that stomatal factors limited the net photosynthetic rate. For example, older leaves serve as the salt-accumulating hotspots under salinity stress. Because of high salt concentrations, the leaf senescence took place prematurely, which lead to a reduction in the photosynthetic leaf area of a plant. Consequently, the photosynthesis rate decreased [12], which led to a lower biomass [59].

A further stress enhancement resulted in a situation when the plant could not tolerate the combination of salinity and drought (i.e., 15 dS m^−1^+Drought treatment) in our study (Figure 3A), that might be due to the extra inclusion of saline ions by plants in a bid for osmotic adjustment, which leads to the ionic imbalance or toxicity. Hence, the closing of stomata may be regarded as an approach to avoid a water deficit (or drought), leading to reduced C assimilation [59,60]. 

For instance, a decline in photosynthetic and transpiration rates was observed in fruit crops with increasing salt stress; and that reduction was mainly attributed to the stomata closing [61,62]. Moreover, in excessive salinity, there was relatively less entrance of CO_2_ into the leaves, which resulted in a reduced photosynthetic rate [63].

Combined stresses such as salinity and drought reduce the water contents in soil, which further reduces soil water and osmotic potential [64]. Moreover, high salt contents in the soil solution hampered the ability of the plant to absorb water and reduced the turgor pressure of leaves [65] and ultimately limited the transpiration rate [12]. The decline in the transpiration rate reduced the ion uptake by roots, xylem conductivity, and decreased ions in leaves [66].

The combined stressors, i.e., salt and drought, decreased the conductance of stomata, due to the inability of the plant to extract water from the soil. Thus, an imbalance was developed between the uptake of water by roots and water lost by transpiration, which consequently resulted in the wilting of the plant [67]. While being exposed to salinity and drought, the plant closes the stomata and avoids dehydration. However, at the same time, the stomata closure also limits the CO_2_ and O_2_ exchange between the atmosphere and internal tissue. Again, this slows down many metabolism-related mechanisms and may reduce plants’ survival rate [68].

## 4. Materials and Methods 

### 4.1. Experimental Design and Crop Establishment

The present study was performed as a pot experiment in a wire-house of the Institute of Soil and Environmental Sciences (ISES), University of Agriculture, Faisalabad (UAF), Pakistan (latitude 31.42° N, longitude 72.08° E, Elevation 187 m). The area is characterized by a mean annual temperature of 24.2 °C and mean annual precipitation of 346 mm [69]. Before the experiment, soil sampling was performed from the research area of the ISES, UAF. Parameters such as pH, ECe, SAR, soluble cations (Na^+^, K^+^), anions (CO_3_^2−^, HCO_3_^−^), and texture were analyzed using standard methods mentioned in USDA Handbook# 60 [70]. Before sieving through a 2-mm sieve, the soil was dried first, and then 8 kg of that soil was poured into each plastic pot (i.e., 25 cm in diameter and 23 cm high).

Non-saline treatment (without NaCl and drought stress) was considered as the control (normal irrigation ~ 1000 mL). Three levels of salinity (7.5, 15, 22.5 dS m^−1^ NaCl), one level of drought (250 mL), and three combined levels of salinity and drought (7.5 dS m^−1^ NaCl+Drought, 15 dS m^−1^ NaCl+Drought, and 22.5 dS m^−1^ NaCl+Drought) were made by mixing NaCl (computed amounts). Well-watered plants were irrigated to 70% of the soil’s water-holding capacity (WHC), and drought stressed plants were irrigated to 40% WHC. Each treatment has five replications and was arranged in a completely randomized design (CRD). Healthy and uniform seedlings (60 days old) were planted in all the pots (one seedling per pot). Tap water was used to irrigate plants.

The basic soil physico-chemical properties were determined before planting the seedlings into the pots. The soil texture was silt loam with 23.5% clay, pH 7.7, EC 2.25 dS m^−1^, SAR 54.1 (mmol L^−1^)^1/2^, bicarbonates were 0.22 mg L^−1^, and carbonates were absent.

### 4.2. Measurement of Growth and Chemical Attributes

Plant growth data for height (PH), stem diameter (SD), and the number of leaves (NOL) and branches (NOB), shoot fresh weight (SFW) and shoot dry weight (SDW), root fresh weight (RFW) and root dry weight (RDW) were quantified three times (after every three months) before the harvesting. A meter tape was used to measure the plant height (cm) from the base to the top of the plant. Vernier calipers were used to record the stem diameter of each plant (5 cm) above the soil surface. 

Plant shoots were excised, and SFW of each plant was weighed at once, and the plants were covered with paper bags and left in the oven to dry at 60 °C for three days. After drying, shoot and root samples were ground separately, shoot and root dry weights (DW) were measured, followed by the measurements of sodium (Na^+^), potassium (K^+^), which were measured using a flame photometer (BWB-XP5). The K^+^: Na^+^ ratios were computed in shoot and root samples. 

### 4.3. Determination of Physiological Attributes

Physiological parameters such as chlorophyll contents of the second leaf were determined with a chlorophyll meter (SPAD-502, Konica Minolta Japan). Prior to harvesting, a portable Infrared Gas Analyzer (IRGA LCA-4 ADC) (Analytical Development Company, Hoddesdon, England) was used to determine photosynthetic and transpiration rates and the stomatal conductance of *Jatropha* plants. All the measurements were made between 11:00 am and 2:30 pm with the adjustments mentioned in Supplementary Material (Table S1). 

Membrane stability index (MSI) was measured following the method proposed by Sairam et al. [71] by analyzing the EC of leaf ions leaked in ultra-pure deionized water. About 100 mg of the leaf sample was taken in a test tube containing 10 ml of ultra-pure deionized water in 2 parts; the first was placed in a water bath for 30 min (40 °C), while the other was placed in a water bath (100 °C boiled water for 15 min), and their EC values (*C*1 and *C*2) were noted, respectively, with an EC meter (HANNA, 99301, Hanna Inst. Inc. RI, USA). The following formula was used for the calculation of the membrane stability index:

$$MSI=\left[1-\left(\frac{C1}{C2}\right)\right]\times 100$$

For the *RWC* determination, a pair of leaves were cut from each shoot, and their *FW* was recorded at once. Then, overnight, they were left floating in distilled water (at 4 °C), and the resaturated weight (*RW*) was measured. Then they were put in the oven at 70 °C overnight for drying and weighed again for the dry weight (*DW*) measurement. The following formula given by Teulat et al. [72] was employed to calculate the *RWC*:

$$RWC\left(\%\right)=\frac{FW-DW}{RW-DW}\times 100.$$

### 4.4. Quantification of Stress Tolerance

#### 4.4.1. Stress Response Models and Salinity-Tolerance Index

Yield response variables such as maximum yield (Y_m_) and relative yield (Y_r_) were studied by Steppuhn [21]. Total plant dry mass (shoots and roots) was expressed as yield (Y), and it was converted to Y_r_ by using a dividing factor Y_m_ that was dependent on the total biomass, which is independent of salinity. The following equation was used to compute Y_r_ value at each salinity level.

(1) Yr=YYm.

On the basis of the best-fitted results and maximum R^2^ values, an exponential model, was used to analyze the yield response to salinity after transforming the data in Equation (1):

(2) $$Y_{r}=a\times e^{b\times ECi}$$

where the electrical conductivity of irrigation water is expressed as *ECi*; a and b are the constants; the former depicts the curve shape, while the latter determines the model intensity. The Salinity Tolerance Index (STI) indicates the inherent ability of crops to tolerate root-zone salinity by Steppuhn et al. [28]. EC_i50_ can be computed from Equation (2); it is the value of EC at which the yield was reduced to 50% of the maximum yield. The STI can be determined as suggested by Steppuhn et al. [73]:

(3) $$\mathrm{ST}-\mathrm{index}={\mathrm{EC}}_{i50}\times \left(1+b\right)$$

#### 4.4.2. Stress Tolerance Determination

The stress tolerance was proposed for yield-related attributes. Here, we have adapted the stress tolerance (with slight modifications) for the plant height from Fernandez [74]. It defines the plant’s ability to maintain sufficient growth in the stress environment corresponding to the control conditions in terms of the overall performance of the plant. The stress tolerance was determined using the following formula:

(4) $$Stress\;Tolerance\;\left(\%\right)=\;\frac{Y_{control}}{Y_{avg}}\;\times \;\frac{Y_{salinity,drought}}{Y_{avg}}\times \;100$$

where *Y* in Equation (4) represents growth-related parameters, e.g., plant height, stem diameter, and total dry mass-produced. *Y_avg_* denotes the average of all the plants under control conditions for plant height *Y*, stem diameter, and total dry mass-produced.

### 4.5. Statistical Analyses

Preliminary data was processed in MS Excel 2016 (for Windows). Data for various parameters were performed by computing one-way Analysis of Variance (ANOVA) utilizing the software SPSS 20.0 Statistical Package for Windows (IBM, Chicago, IL, USA), followed by Duncan’s multiple comparisons post-hoc test to determine the least significant differences between treatment means. Differences in treatment means were expressed as the standard error (±SE), see Steel et al. [75]. The ANOVA and Duncan tests were both set at *p* < 0.05. 

## 5. Conclusions

In summary, the data presented in this study demonstrate that *J. curcas* can be considered to be a more tolerant crop if the stressors are not combined. However, the combined stresses of salinity and drought (no matter if the salinity level was low or high) created more menace in less biomass production, due to decreased tolerance and disturbed physiological mechanisms. Plants treated with 15 dS m^−1^ NaCl showed a significant reduction in growth. The MSI and RWC contributed the most towards stress tolerance. Thus, it could easily be grown in saline soils of arid to semi-arid regions, albeit prospective work regarding assays in field conditions is imperative for furthering insight into biomass production and quality. 

## Figures and Tables

**Table 1:** The effect of salinity and drought stress on growth and physical parameters of *Jatropha curcas.*

Treatments	Plant Height (cm)			Stem Diameter (cm)			No. of Branches			No. of Leaves		
	Duration (months)			Duration (months)			Duration (months)			Duration (months)		
	3	6	9	3	6	9	3	6	9	3	6	9
Control	62.6 ± 0.93a	68.80 ± 0.86a	72.00 ± 1.58a	1.82 ± 0.06d	1.93 ± 0.03a	2.40 ± 0.18a	12.20 ± 0.37a	14.80 ± 0.86a	16.40 ± 0.86a	11.40 ± 0.68a	14.20 ± 0.86a	15.80 ± 0.86a
Drought	33.4 ± 1.81b	36.20 ± 1.74b	41.40 ± 1.58b	1.07 ± 0.06bc	1.20 ± 0.11c	1.37 ± 0.05bc	9.80 ± 0.37b	12.20 ± 0.73b	13.60 ± 0.51b	7.40 ± 0.81b	10.20 ± 0.37b	13.20 ± 0.58b
7.5 dS m^−1^ (Well-watered	21.00 ± 1.00c	22.60 ± 1.03c	26.80 ± 0.97c	1.58 ± 0.08b	1.63± 0.08b	1.68 ± 0.07b	9.60 ± 0.68b	10.80 ± 0.86b	13.20 ± 1.02bc	7.20 ± 0.80b	10.20 ± 0.86b	12.40 ± 1.33b
7.5 dS m^−1^+Drought	13.85 ± 1.92d	14.67 ± 0.93d	17.20 ± 1.58d	1.04 ± 0.07c	1.15 ± 0.04c	1.48 ± 0.19bc	7.20 ± 0.66c	8.00 ± 0.55c	11.20 ± 0.37cd	6.60 ± 0.51b	7.60 ± 0.51c	9.40 ± 0.51c
15 dS m^−1^ (Well-watered)	12.89 ± 1.07d	15.81 ± 0.97d	18.63 ± 1.58d	1.08 ± 0.06c	1.14 ± 0.06c	1.16 ± 0.01cd	6.80 ± 0.58c	7.20 ± 0.66cd	10.00 ± 0.71d	6.20 ± 0.58b	6.80 ± 0.66c	7.60 ± 0.51cd
15 dS m^−1^+Drought	10.75 ± 1.95d	13.70 ± 0.93d	16.44 ± 1.58d	0.56 ± 0.08d	0.84 ± 0.07d	1.08 ± 0.06d	4.80 ± 0.97d	5.20 ± 0.37d	7.60 ± 0.75e	4.00 ± 0.55c	4.40 ± 0.51d	5.60 ± 0.51e

Data are the mean values ± SE (*n* = 5). Different lowercase letters within a column indicate significant differences among treatments at *p* < 0.05, according to Duncan’s least significance test. Note: Well-watered means plants irrigated to 70% of soil water holding capacity (WHC), while Drought means plants were irrigated to 40% of soil WHC.

**Table 2:** Concentrations of Na^+^, K^+^, and K^+^: Na^+^ ratio in *Jatropha curcas* plants subjected to the salt and drought stresses induced by NaCl and low water supply, respectively. Data refer to mean values (*n* = 5).

Treatment	Na^+^			K^+^			K^+^: Na^+^ Ratio		
	Leaf	Stem	Root	Leaf	Stem	Root	Leaf	Stem	Root
Control	0.12e	0.14e	0.16e	1.62a	0.68a	0.55a	13.06a	4.72a	3.49a
7.5 dS m^−1^ (well-watered)	0.60d	0.66d	0.79d	0.89c	0.48c	0.41b	1.483c	0.73c	0.52c
15 dS m^−1^ (well-watered)	1.29b	1.51b	1.89b	0.83c	0.43cd	0.38b	0.643c	0.29c	0.20c
Drought	0.16e	0.18e	0.19e	1.24b	0.57b	0.49a	7.949b	3.28b	2.53b
7.5 dS m^−1^+Drought	1.05c	1.12c	1.25c	0.87c	0.46c	0.39b	0.829c	0.41c	0.31c
15 dS m^−1^+Drought	1.84a	2.06a	2.32a	0.75d	0.36d	0.27c	0.408c	0.18c	0.12c
									

Different lowercase letters within a column depict significant differences among treatments, according to Duncan’s Least Significance Test after ANOVA (*p* < 0.05). Note: Well-watered means plants irrigated to 70% of soil water holding capacity (WHC), while Drought means plants were irrigated to 40% of soil WHC.

## Supplementary Materials

The following are available online at www.mdpi.com/xxx/s1, Table S1.
