# Supplementary material for: Evaluating the Contribution of Growth, Physiological, and Ionic Components Towards Salinity and Drought Stress Tolerance in Jatropha curcas
Source: Plants (Basel). 2020 Nov 13;9(11):1574. doi: 10.3390/plants9111574 (PMC7696781; doi:10.3390/plants9111574)
Supplement: Supplementary file 1 [file plants-09-01574-s001.pdf]

Table 1 Adjustments made for IRGA to measure photosynthetic and transpiration rates, and stomatal conductance of all Jatropha plants

**Sr.#**	**Specifications**	**Values**
1.	Leaf chamber temp.	34.6-39.5 °C
2.	ambient pressure	990 mBar
3.	Ambient CO_2 concentration (C_{ref})	373 µmol mol^{-1}
4.	sub-stomatal CO_2 concentration (Ci)	115-312 µmol mol^{-1}
5.	average water vapor pressure into chamber	34.6 mBar
6.	molar flow of air per unit leaf area (Us)	206.6 mol $m^{-2}s^{-1}$
7.	PAR (Q leaf) at the leaf surface	1200 µmol $m^{-2}s^{-1}$
